# The RNA-centred view of the synapse: non-coding RNAs and synaptic plasticity

**DOI:** 10.1098/rstb.2013.0504

**Published:** 2014-09-26

**Authors:** Neil R. Smalheiser

**Affiliations:** Department of Psychiatry, University of Illinois at Chicago, Chicago, IL 60612, USA

**Keywords:** microRNAs, non-coding RNAs, synaptic plasticity, transposable elements

## Abstract

If mRNAs were the only RNAs made by a neuron, there would be a simple mapping of mRNAs to proteins. However, microRNAs and other non-coding RNAs (ncRNAs; endo-siRNAs, piRNAs, BC1, BC200, antisense and long ncRNAs, repeat-related transcripts, etc.) regulate mRNAs via effects on protein translation as well as transcriptional and epigenetic mechanisms. Not only are genes ON or OFF, but their ability to be translated can be turned ON or OFF at the level of synapses, supporting an enormous increase in information capacity. Here, I review evidence that ncRNAs are expressed pervasively within dendrites in mammalian brain; that some are activity-dependent and highly enriched near synapses; and that synaptic ncRNAs participate in plasticity responses including learning and memory. Ultimately, ncRNAs can be viewed as the post-it notes of the neuron. They have no literal meaning of their own, but derive their functions from where (and to what) they are stuck. This may explain, in part, why ncRNAs differ so dramatically from protein-coding genes, both in terms of the usual indicators of functionality and in terms of evolutionary constraints. ncRNAs do not appear to be direct mediators of synaptic transmission in the manner of neurotransmitters or receptors, yet they orchestrate synaptic plasticity—and may drive species-specific changes in cognition.

## 1. Introduction

A significant subset of mRNAs are differentially transported and translated in dendrites and in proximity to individual synapses [1]. If mRNAs were the only RNAs made by a neuron, there would be a simple mapping of mRNAs to proteins. However, microRNAs (miRNAs) and other non-coding RNAs (ncRNAs) regulate gene expression via effects on protein translation as well as transcriptional and epigenetic mechanisms [2]. These additional classes of RNAs, and the additional layers of regulation that they perform, make the pattern of ncRNA expression distinct from the pattern of gene expression *per se*—as not only can genes be ON or OFF, but their ability to be induced or translated can be turned ON or OFF as well. As we shall see, these decisions can be made locally at the level of synapses.

In this paper, I shall review evidence that ncRNAs are expressed pervasively within dendrites in mammalian brain; that subsets of ncRNAs are activity-dependent and highly enriched near synapses; and that these synaptic ncRNAs participate in plasticity responses including learning and memory. My intent is not merely to summarize current evidence, but to point out gaps, discuss areas of neglect and controversy, synthesize underlying principles and suggest promising lines of investigation for the future.

## 2. Dendrites and protein synthesis

An early seminal clue to the molecular basis of learning was the finding that protein synthesis inhibitors block the acquisition of learning within critical time windows [3,4]. It was uncertain whether general protein synthesis is required (as a permissive function) or whether synthesis of particular proteins is needed (as an instructive signal). However, this controversy became less relevant once it was discovered that discrete sites of protein synthesis occur in different parts of the dendritic tree, since even general or ‘housekeeping’ protein synthesis of cytoplasm can have an instructive role if it supports widening of a particular dendritic branch or growth of a particular dendritic spine. Polyribosomes are localized within dendritic shafts, and at the base of dendritic spines; after intense synaptic activity, polyribosomes are seen to invade spines and reside in proximity to the synapse at the postsynaptic density (PSD) [5–7]. Proteins that regulate translation, including initiation and elongation factors, have also been localized in proximity to postsynaptic densities [8]. This correlates with the identification of a microtubule-based motor system that moves RNA transport granules into dendritic shafts and an actin-based motor that is involved in transport into dendritic spines. The transport of at least some mRNAs into dendrites is stimulated by activity [1].

At one time, it was thought that only a handful of mRNAs were transported to dendrites. However, selective dendritic transport is now known to occur for hundreds of different mRNAs, including those with well-documented plasticity functions (e.g. fragile X mental retardation protein (FMRP), Ca^2+^/calmodulin-dependent protein kinase II alpha (CaMKIIα), postsynaptic density protein 95 (PSD-95) and matrix metallopeptidase 9 (MMP9)), that are translated locally in an activity-dependent manner. A variety of mRNA- and miRNA-containing structures have been observed within dendrites, including P bodies, stress granules and other poorly characterized complexes [9], at least some of which undergo dissolution following synaptic activity in parallel with the de-repression of protein translation. Not all mRNAs within the neuron are targeted for dendrites (e.g. members of a different subpopulation are targeted down axons), but roughly 15% of all mRNAs made by a neuron seem to have dendritic roles [1]. Fragile X syndrome (FXS), a genetic disease of intellectual disability in which FMRP is not expressed, causes over-translation of proteins to occur in neurons and isolated synaptic fractions under basal conditions [10]. FMRP binds a subset of synaptic mRNAs and represses their translation. Treatments which normalize the protein synthesis rates in mouse models of FXS also revert some of the behavioural deficits [10]. All of the available evidence suggests that protein synthesis plays a role in learning, both via supporting long-term changes in growth and branching of dendritic spines, shafts and axonal arborizations, and by pathways that induce specific synaptic proteins [11].

## 3. The post-genomic world of non-coding RNAs

The process of protein synthesis starts with transcription of primary mRNA precursors (pre-mRNAs) containing 5′-caps and poly A+-tails which generally undergo splicing in the nucleus to remove introns, and then are transported into the cytoplasm as mature mRNAs where they may be stored or translated. mRNA translation into proteins can be regulated at the level of initiation, elongation or termination. Abundant ncRNAs participate in the canonical steps in translation (e.g. U RNAs in spliceosomes, rRNA in ribosomes, tRNAs binding amino acids and small nucleolar RNAs (snoRNAs) involved in the maturation of these ncRNAs).

Surprisingly, the post-genomic revolution has revealed that the majority of RNAs expressed by cells are not protein-coding mRNAs, but rather ncRNAs [12] (table 1). These include small RNAs—miRNAs, endogenous small inhibitory RNAs (endo-siRNAs), piwi-interacting RNAs (piRNAs), transposable element (TE)- and repeat-derived small RNAs and small RNAs derived from abundant ncRNAs—as well as longer RNAs such as antisense transcripts, long intronic and intergenic ncRNAs, and TE- and repeat-related transcripts [13,14]. Circular RNAs appear to act, in part, as natural miRNA sponges [15,16]. Most, if not all, of the ncRNA classes have been shown to regulate gene expression via post-transcriptional actions on mRNA stability and/or translation. They also regulate transcriptional activation or repression of specific genes and epigenetic modifications of chromatin [2,13,14].

**Table 1.** The world of ncRNAs. ncRNAs discussed in this review, organized by approximate size ranges in nucleotides. (The full list of ncRNAs includes ultra-short RNAs (less than 18 nt), satellite-associated RNAs, promoter-associated RNAs, half tRNAs and others.)

18–24	miRNAs; endo-siRNAs
18–40	small RNAs processed from abundant cellular ncRNAs; small RNAs processed from TE transcripts
25–30	small RNAs processed from abundant cellular ncRNAs (pincRNAs) that are specifically regulated by learning
25–35	typical piRNAs expressed in the germline (and in somatic tissues at lower levels)
70–110	pre-miRNAs (miRNA small hairpin precursors)
70–300	abundant cellular ncRNAs: tRNAs, snoRNAs, Y RNAs, vault RNAs, snRNAs and 5S rRNA
100–300	Alu-related RNA transcripts; BC1; BC200
>300	long intronic and intergenic ncRNAs, pri-miRs (primary miRNA gene transcripts), antisense RNAs, circular RNAs, TE transcripts, 18S and 28S ribosomal RNAs

## 4. First clues that non-coding RNAs are pervasive within dendrites

Several recent reviews have emphasized that miRNAs and other ncRNAs play roles in plasticity processes related to learning, memory and neuropsychiatric diseases [2,17–23]. However, I do not believe that it is appreciated just how deeply the world of ncRNAs is situated within the synaptic compartment.

The first clue that regulatory functions (and RNAs) thought to be restricted to the nucleus might actually be available for local regulation near synapses came from Eberwine and colleagues. They demonstrated that primary messenger RNA gene transcripts (pre-mRNAs) containing retained introns are not all processed within the nucleus, but can be transported into dendrites, where they can undergo alternative splicing to generate protein variants having distinct functional properties that affect membrane excitability [24–27].

**Figure 1.**
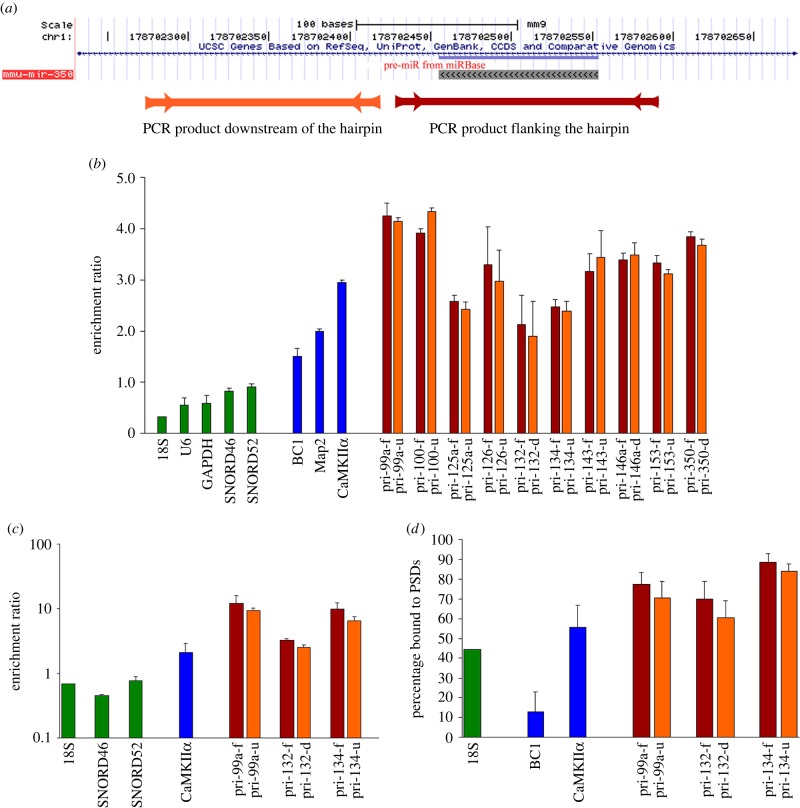
Pri-miRs are enriched within synaptoneurosomes, synaptosomes and isolated PSDs. (*a*) Diagram showing the genomic position of pre-miR-350 in the UCSC Genome Browser, indicating the PCR products flanking the hairpin (Bordeaux (darker) bar) and downstream of the hairpin (orange (lighter) bar). (*b*) Enrichment ratio (synaptoneurosomes/total homogenate) of various RNAs as measured by qRT-PCR. (*c*) Enrichment ratio (synaptosome/total homogenate) of intergenic pri-miRNAs measured by qRT-PCR. Note the log scale. (*d*) Distribution of pri-miRNAs and control RNAs in synaptosomes (soluble versus PSD fractions). Synaptosomes (Syn) were lysed with 1% Triton X-100 to yield soluble (Sol) and insoluble fractions (PSD). (*b*–*d*) Housekeeping RNAs and known synaptic RNAs are indicated by green bars. PCR products flanking the hairpin are indicated by Bordeaux (darker) bars and the PCR products either downstream or upstream of the hairpin are indicated by orange (lighter) bars. Data represent the geometric mean of three independent preparations (±s.e.m.). To minimize differences across preparations, values were normalized to 18S using the delta-delta ΔΔC_t_ method. Reprinted from [28] with permission. (Online version in colour.)

Recently, my laboratory obtained evidence that primary miRNA gene transcripts (pri-miRs) are not all processed within the nucleus, as expected from prevailing models of miRNA biogenesis [28]. Instead, pri-miRs are also expressed in cytoplasmic fractions enriched for RNA transport granules, where they are directly associated with KIF5 heavy chain, a motor protein used for dendritic RNA transport. Furthermore, the pri-miRs are tightly associated with drosha and DGCR8, the enzymes that process the pri-miR to small hairpin precursors (pre-miRs). All of these components (pri-miRs, drosha and DGCR8) are enriched in purified synaptic fractions (synaptosomes and synaptoneurosomes) and isolated PSDs [28] (figure 1). This is true both for intronic miRNAs (which reside within retained introns of pre-mRNAs) and intergenic miRNAs (whose pri-miRs are freestanding long ncRNAs in their own right).

In fact, the entire machinery for miRNA biogenesis can be localized to dendritic spines [29,30]. Dicer, the enzyme that processes pre-miRs to mature miRNAs, is enriched at PSDs as shown by electron microscopic immunocytochemistry of mouse hippocampal neurons [29]. When isolated PSDs are examined, full-length dicer protein is present but in an enzymatically inactive state; incubation with calpain causes cleavage of dicer into smaller fragments that have highly active RNAse III activity [29]. When acute hippocampal slices are stimulated with *N*-methyl-d-aspartate receptor (NMDA), specific dicer fragments are formed in a manner that depends upon calcium and calpain activation [29]. The majority of neuronally expressed miRNAs, along with the core component of the RNA-induced silencing complex (RISC), Ago2, are expressed in synaptic fractions [29,30]. miRNAs in dendrites appear to regulate mRNAs in much the same way as they do in the cell body, i.e. by binding to target mRNAs and exhibiting a negative influence on their translation and/or stability [17–21].

A subset (approx. 15%) of miRNAs is significantly enriched in synaptic fractions (up to approx. fivefold) compared with total tissue homogenate [30]; these differ from the majority of neuronal miRNAs in their precursor structure, evolutionary history and level of expression [30,31]. (Within hippocampus as a whole, the population of synaptically enriched miRNAs is expressed at lower levels as compared with those which are not enriched—yet synaptically enriched and depleted miRNA subgroups are expressed at the same absolute levels within synaptic fractions, so both populations are likely to be functional within the synaptic compartment [31].) Although it still remains to be shown directly that pri-miRs are converted to mature miRNAs within individual dendritic spines, the synaptic enrichment ratio of selected miRNAs correlates well with the enrichment ratio of their precursors [30]. These findings all suggest that miRNAs are formed (at least in part) locally, in response to signals arising at individual synapses.

Many of the individual proteins associated with mRNA transport also regulate steps in alternative splicing and in miRNA processing. To give a few examples, EWS, TLS/FUS, TDP-43 and DDX5/p68 all associate with drosha [32]; hnRNP A1 binds and regulates pri-miR-18a [33]; Translin binds certain miRNAs [34] and C3PO (Translin/Trax complex) participates in loading of the RISC complex [35]. As well, Huntingtin binds Ago2, and FMRP binds to multiple components of the miRNA pathway including dicer, Ago2, mature miRNAs and miRNA precursors [36,37]. Dendritic transport and processing of mRNAs, pre-mRNAs and pri-miRs may comprise a single integrated process.

In any case, it is appropriate to regard the synaptic compartment as a relatively independent, self-contained arena for RNAs, because several activity or learning paradigms cause a pattern of miRNA changes that is quite different when measured within isolated synaptic fractions versus when measured within whole tissue homogenates [38–40]. This point is underscored by our recent report of miRNA expression in human post-mortem prefrontal cortex in schizophrenia [41]. When whole tissue was examined, we observed that 13 miRNAs were significantly upregulated in schizophrenia subjects versus six miRNAs that were downregulated. The upregulated miRNAs include a module that shared 5′- and 3′-seed sequences, as well as miRNAs known to be enriched in white matter (none are brain-enriched, and only two of the 13 upregulated miRNAs have synaptic enrichment ratios of more than 1.5). By contrast, five of the six downregulated miRNAs have synaptic enrichment ratios of 1.5 or greater [41]. This suggested that the downregulation might selectively affect synaptic miRNAs. Indeed, isolated synaptosomes prepared from these samples show a large, global downregulation (significant for 73 miRNAs), and those miRNAs which are the most highly synaptically enriched show the greatest extent of downregulation [41]. As a control, another class of RNAs of the same size (derived from H/ACA or C/D box snoRNAs) does not show any alteration in this dataset; this shows that the downregulation is not an artefact of sample preparation or normalization [41]. These findings point to some deficit in miRNA biogenesis, transport, processing or turnover in schizophrenia that is selective for the synaptic compartment.

If retained introns and pri-miRs are present within dendrites, then this raises the question of whether another function normally associated with the nucleus—RNA editing—might also occur locally within dendrites. To my knowledge, no one has investigated that question, but that would be worth exploring since ADARs often edit miRNA precursors and intronic sequences and some ADAR1 isoforms are expressed in the cytoplasm [42,43].

## 5. MicroRNAs: modular and global effects

Among the ncRNAs, the miRNAs have been far the best investigated in terms of their roles in plasticity processes. Several reviews have summarized evidence that miRNAs are modulated by synaptic activity or brain-derived neurotrophic factor (BDNF) and, in turn, regulate activity-dependent protein synthesis, dendritic spine morphogenesis, axonal outgrowth, and learning and memory [2,17–23]. The miRNAs are often regulated in modules or groups: for example, a subset of miRNA genes has cAMP response elements (CREs) in their promoters and their transcription is induced by cAMP response element binding protein (CREB). Synaptic activity affects translation via several signalling pathways (mTOR, ERK, eEF2 and others) which modulate, and are modulated by, miRNAs [22,23].

Here, I will focus on the possible significance of global alterations in miRNA expression, which in my opinion has been relatively neglected. Global changes measured in high-throughput experiments are susceptible to technical artefacts, which has led many investigators to carry out normalization of miRNA values in ways that remove the ability to detect global changes. However, it is possible to rule out artefacts with the use of controls including exogenous spike-in RNAs, appropriate endogenous housekeeping RNAs and contrasting changes among different types and size classes of RNAs in the same sample [41,44,45]. The cancer field has increasingly focused on global alterations of miRNA expression, and altered levels of drosha, dicer and other biogenesis components have been correlated with tumour type and progression [46]. Enoxacin, an agent that causes global upregulation of miRNA expression via binding *trans*-activation response RNA-binding protein (TRBP) and stabilizing dicer activity [47], appears to revert cancer phenotypes both in cultured cells and *in vivo* animal models [48].

Several plasticity paradigms affect miRNAs globally: (i) BDNF stimulates the translation of dicer in cultured hippocampal neurons [49]. This elevates miRNAs generally which represses protein synthesis globally. However, BDNF also induces lin28 which binds to loop sequences present in a subset of pre-miRs and inhibits their processing, thus selectively de-repressing the translation of their target mRNAs [49]. (ii) Hippocampal slices subjected to chemical long-term potentiation (LTP) show an upregulation of almost all measured miRNAs at 30 min [50]. (iii) miRNAs are globally upregulated at an early phase of acquisition of two-odour olfactory discrimination training in adult mouse hippocampus [44]. (iv) In hippocampus of rats subjected to status epilepticus for 4 h, there is a significant increase in 67 miRNAs and none that decreased, whereas most miRNAs are downregulated at 48 h [38].

The mechanism(s) for upregulation in these paradigms are unknown but may include, e.g. stimulating dicer translation [49], activating dicer protein via calpain-mediated cleavage [29], phosphorylating TRBP [51] or phosphorylating drosha. Global upregulation of miRNAs is likely to dampen down the burst of protein synthesis that follows synaptic stimulation with mGLuR5 or NMDA receptors [10,49]. As excessive tonic protein synthesis is a feature of FXS, and reversing this appears to rescue some of the fragile X disease phenotypes in mice and cultured cells, miRNAs may be playing a role similar to FMRP (indeed the two may be working together in this pathway, given the close interaction of FMRP with miRNA components, and the observation that phosphorylation of FMRP prevents its binding to dicer [52]). Downregulation might be related to loss of transcription or processing of precursors, or turnover of miRNAs or RISC complexes [53,54]. The degree of association between miRNAs and Ago is subject to regulation, which may alter the effectiveness of miRNAs even in the absence of changes in their abundance [55]. Selective cleavage of RISC components, e.g. Armitage or MOV10, has been reported to be a necessary event in learning and activity paradigms and de-represses miRNA-mediated inhibition of their target mRNAs [56,57].

Several studies have engineered mice that are deficient in miRNA biogenesis components, which decrease expression of the vast majority of miRNAs. Dicer knockout in forebrain neurons increases levels of synaptic proteins and enhances learning and memory [58]; however, it is unclear whether this effect relates to changes in individual key miRNAs, global changes in miRNA abundance or changes in other classes of small ncRNAs that are processed by dicer. DGCR8 heterozygosity, which affects drosha-dependent miRNAs, hurts performance in the Morris water maze [59] as well as producing discrete effects on neuronal excitability, dendritic trees and neurogenesis [60,61]. Hsu *et al*. [62] produced a conditional knockout of DGCR8 in postnatal forebrain neurons and observed a non-cell-autonomous reduction in parvalbumin interneurons in the prefrontal cortex, accompanied by a severe deficit in inhibitory synaptic transmission and a corresponding reduction of inhibitory synapses. We recently reported that there is a global downregulation of miRNA expression in human post-mortem prefrontal cortex (whole tissue homogenates) in depressed suicide subjects [45] and replicated this finding in a second suicide cohort [41]. Interestingly, enoxacin pre-treatment of rats for one week raises miRNA levels in frontal cortex and prevents learned helplessness following inescapable shock, a rodent model of depressive behaviour [63]. These findings raise the possibility that global alterations in miRNA levels may not only relate to neuropsychiatric disorders but may be a promising therapeutic target.

## 6. RNA interference and learning

RNA interference (RNAi) is a sequence-specific phenomenon in which a small RNA (approx. 18–24 nt), complexed with an Argonaute (Ago) family homologue, binds in perfect or near-perfect complementarity to a target RNA and activates a ‘slicer’ activity in the Argonaute protein that cuts the target RNA at a specific site [64]. (This cut is generally presumed to destabilize the target RNA and lead to its rapid destruction, though cuts might serve a processing or biosynthetic function in some cases.) The 25–35 nt piRNAs associate with Piwi homologues (members of the Argonaute super-family), and the piwi/piRNA complexes also affect target RNA stability and translation, so despite differences in their biogenesis and biology, piRNAs can be considered to be a variant pathway of RNAi [65,66].

A variety of cellular RNAs, having double-stranded character or that contain hairpin secondary structures, can be processed by dicer to generate so-called endo-siRNAs that are approximately 22 nt long and that associate with Ago2 in mammalian cells to mediate RNAi. Endo-siRNAs can arise from sense–antisense RNA hybrids, pseudogene transcripts, TE transcripts, or mRNA exons or introns that fold into hairpin secondary structures [67,68]. (miRNAs can also mediate RNAi if they are a perfect match to their targets.)

As originally characterized in *Caenorhabditis elegans*, RNAi exhibits a number of striking, even amazing features: (i) RNAi is not only extremely potent, but it has a self-amplifying and self-propagating nature. This is because a siRNA binding a long target RNA can act as a primer for extension by an enzyme, RNA-dependent RNA polymerase (rdrp), which creates a long second strand. This double-stranded target can now be processed by dicer to form secondary siRNAs. Furthermore, the secondary siRNAs derive from multiple sites along the target which increases the overall potency and magnitude of the response. (ii) RNAi silencing of one tissue can spread systemically throughout the entire body, including the germline. (iii) Exogenous double-stranded RNAs can be taken up by the gut and processed to form siRNAs that mediate effective silencing. In 2001, my colleagues H. Manev, E. Costa and I pointed out [69] that the properties of RNAi in *C. elegans* are surprisingly similar to the properties of ‘memory transfer’ in planarians (flatworms) as reported in a series of controversial studies by McConnell and co-workers [70,71].

McConnell reported that planarians could be reliably conditioned to turn in response to light or vibration. Taking advantage of the regenerative capacity of planarians, he separated the head (containing the brain) from the tail in trained animals and reported that persistent behavioural changes were seen in animals that regenerated from either half. Furthermore, conditioning was enhanced by injecting extracts of trained planarians into naive planarians, or (because planarians are cannibals) even just feeding them trained animals. Tellingly, the active principle in the extract appeared to be RNA [70,71]. Putting this together, we suggested that some RNAi signal may have been generated during learning in the flatworm, which spread systemically (hence survived regeneration, tissue extraction and feeding) [69].

In the intervening decade, the case for RNAi-mediated learning in lower organisms has grown stronger: (i) Basic properties of RNAi in planarians appear similar to that in *C. elegans* [72]; (ii) A recent study, employing an automated training paradigm, has confirmed several of McConnell's key findings, namely, that flat worms exhibit environmental familiarization and that this memory persists for at least 14 days—long enough for the brain to regenerate. They further showed that trained, decapitated planarians exhibit evidence of memory retrieval in a savings paradigm after regenerating a new head [73]; (iii) A form of behavioural sensitization in *C. elegans* has been shown to be mediated by RNAi, via a particular class of endo-siRNAs that bind to WAGO (an Argonaute homologue) and that act within the nucleus [74].

Whereas miRNAs have become intensively studied by neuroscientists, there has been virtually no interest in exploring whether RNAi may be a naturally occurring process to regulate long-term gene expression within the mammalian brain [69]. I have recently reviewed this issue in detail [75]. Part of this neglect is due to the prevailing attitude that endo-siRNAs serve to recognize and destroy TEs and other foreign RNAs and should not act physiologically upon a cell's own mRNAs. Another problem is that differentiated mammalian cells may shut down protein synthesis non-specifically when they encounter double-stranded RNAs. Several groups who looked for siRNAs in somatic tissues reported very low expression, which was assumed to be biologically negligible [75].

**Figure 2.**
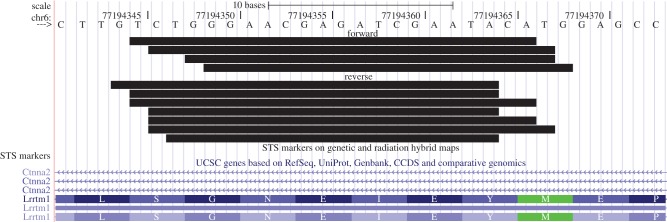
Small RNAs aligned to the *Ctnna2* locus that putatively arise from processing of sense–antisense RNA hybrids. Multiple sequences align to a region of the *Ctnna2* gene that also encodes the *Lrrtm1* locus on the opposite strand. The small RNAs shown here align to both forward and reverse strands and exhibit a high degree of overlap. Reprinted from [76] with permission. (Online version in colour.)

**Figure 3.**
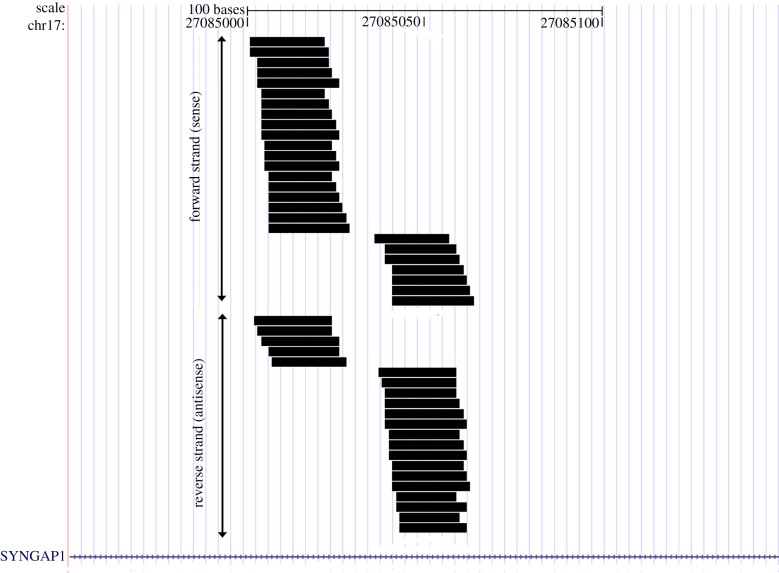
Small RNAs aligned to the *SynGAP1* locus. Shown are all unique sequences that mapped to *SynGAP1*, including those that aligned to the forward or plus strand (placed on top) and to the reverse or minus strand (placed below the forward sequences). Reprinted from [76] with permission. (Online version in colour.)

**Figure 4.**
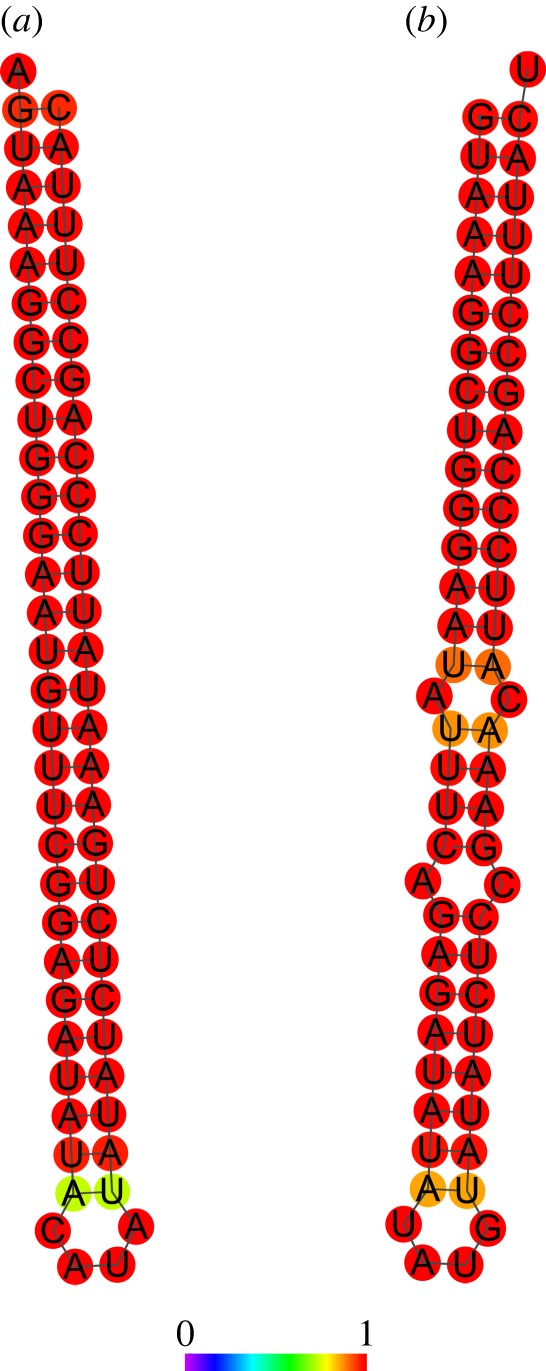
Predicted secondary structure of RNA corresponding to the region within the *SynGAP1* locus that aligns with small RNAs. (*a*) The RNA encoded on the forward strand in the region covered by small RNAs (figure 3) is predicted to form a perfect hairpin inverted repeat. (*b*) RNA encoded on the reverse strand forms an almost-perfect hairpin as well. Colours indicate the probability of base-pairing at each particular residue. Reprinted from [76] with permission. (Online version in colour.)

To look for endo-siRNAs, my laboratory carried out deep-sequencing of RNA extracted from hippocampus of adult mice that were trained on a two-odour olfactory discrimination task. Two negative control groups were used: a naive group that performed nose-poke for water reward but received no odours, and a pseudo-training group that received pairs of odours associated with two ports in randomized fashion but received water reward regardless of odour pairing. We reported learning-associated changes in several classes of small RNAs, including miRNAs [44] and a set of novel ncRNA-derived small RNAs (see §7). However, the deep sequencing data also gave strong expression signatures for several types of endo-siRNAs [76]:

(i) One locus, producing highly overlapping small RNAs in both sense and antisense orientation, resides at a site within the α-N-catenin gene that also encodes the leucine-rich repeat transmembrane neuronal 1 (Lrrtm1) gene on the opposite strand, thus comprising a natural sense–antisense pair of transcripts (figure 2). Both of these genes encode synaptic organizer proteins.
(ii) A set of small RNAs are derived from hairpin secondary structures residing within the introns of eight genes that encode synaptic plasticity-related proteins, including Syngap1 (figures 3 and 4), GAP43, synapsin I and CAMKIIα. Endogenous Syngap1 siRNA was shown to bind to Argonaute in co-immunoprecipitation experiments carried out in brain extracts under stringent conditions, and a synthetic Syngap1 hairpin RNA was shown to be processed by dicer *in vitro* [76].
(iii) Still other small RNAs were detected that have expression signatures suggestive of having been formed by RNAi. For example, we detected small RNAs that aligned to antisense transcripts within the BDNF locus, as well to numerous loci that were previously shown to co-express sense and natural antisense transcript pairs within synaptic fractions [77], including BACE1, SNAP25 and others [76].

These findings show conclusively that endo-siRNAs are expressed in mammalian brain, are increased during an early phase of learning and are linked to synaptic plasticity loci. It is interesting to note that our paper appeared at about the same time as a different deep sequencing analysis of naive mouse hippocampus which did not detect significant expression of endo-siRNAs in brain [78]. Presumably, the windowing and filtering criteria that they employed were not sensitive enough to detect the specific signatures of individual siRNAs associated with synaptic plasticity loci.

RNAi silencing of genes appears to persist for at least several weeks in post-mitotic mammalian neurons [79], which is encouraging in that it may mediate long-term changes in gene expression. No one, to my knowledge, has ever detected, or even looked for, the presence of secondary siRNAs in any mammalian cell type, which would potentially increase the potency, scope and longevity of the RNAi response. However, at least two mammalian sources of rdrp activity have recently been identified (non-canonical activities associated with telomerase [80] and pol II [81]). The major Syngap1 endo-siRNA is expressed in synaptic fractions (G. Lugli and N. Smalheiser 2012, unpublished observations), suggesting that it is worthwhile to examine the possible roles for RNAi not only within mammalian neurons but specifically within the synaptic compartment.

## 7. Small RNAs derived from abundant non-coding RNAs: pincRNAs

During the two-odour olfactory discrimination training experiment discussed in the previous section, many non-miRNA sequences in the size range 18–30 nt were identified that aligned exactly and uniquely to the human genome and that mapped within known gene loci [75,76,82]. These mostly derived from abundant cellular ncRNAs, including snoRNAs, Y RNAs, rRNAs, RMRP and others (small RNAs derived from tRNAs were also well expressed, albeit they generally aligned to more than one genomic location). Intriguingly, a subset of these small RNAs has little or no expression in hippocampus of mice in either control group, but are very strongly induced by training (up to 100-fold or more). These learning-induced RNAs were all 25–30 nt in length (though since 30 nt was the sequencing cut-off, it is unknown at present if longer RNAs may share this effect). Typical miRNAs did not express variant sequences in this size range, and did not participate in this effect, except interestingly, several mirtrons also expressed 25–30 nt sequences that were strongly induced by learning [76].

Lee *et al.* [83] also carried out deep sequencing (up to 35 nt) in hippocampus of naive caged mice and found significant expression of small RNAs in the size range 25–32 nt. The most abundant sequences in their dataset were derived from ncRNAs, and indeed, we and they identified some of the same sequences [76,83]. Lee *et al*. [83] demonstrated that some of these RNAs were associated with MIWI protein (one of the Piwi homologues expressed in mice) and were expressed within dendrites of cultured hippocampal neurons. Antisense inhibition of one of these RNAs in cultured neurons led to a decrease in dendritic spine area, suggesting a role in spine morphogenesis [83]. Although the ncRNA-derived small RNAs appear to bind Piwi homologues, they are not typical piRNAs (e.g. they do not originate from piRNA loci, and do not show a preference for initial U) [75]. I propose referring to small RNAs that derive from abundant ncRNAs (in the size range 25–35 nt) as pincRNAs for the time being, until their relation to typical piRNAs becomes clarified further.

These studies suggest that MIWI/small RNA complexes may be regulating the translation and/or stability of target RNAs involved in dendritic functions. Although the nature of these target(s) remains unclear, it is noteworthy that the host genes for the ncRNAs giving rise to these small RNAs are all 5′-TOP genes, which are activated by mTOR and which include proteins that are integral parts of translation machinery itself (e.g. ribosomal subunits) [76]. The rRNAs, tRNAs, snoRNAs, etc. that are presumably processed to give rise directly to pincRNAs are encoded within introns of these host genes. Thus, one possibility is that pincRNAs locally regulate the expression or function of the ncRNAs and/or their host genes, which participate in mTOR-stimulated protein synthesis. This would allow control over the overall size of, say, a dendritic branch. Interestingly, Kye *et al.* [84] observed that upregulated miRNAs produced by contextual conditioning in mice tended to inhibit inhibitors of the mTOR pathway, suggesting that miRNAs may also participate in mTOR-stimulated general protein synthesis.

Are pincRNAs expressed or enriched in synaptic fractions? Unfortunately, we have not yet examined small RNA expression in synaptic fractions of mice in learning paradigms. However, in unpublished work, I have carried out deep sequencing of hippocampus in naive adult male caged mice covering a size range up to 35 nt, comparing total hippocampal homogenate versus isolated synaptosomes. Many ncRNA-derived small RNAs in the size range 25–35 nt were, indeed, well expressed and a subset was highly enriched in synaptosomes. In fact, among those sequences that were well expressed, 65 distinct sequences exhibited more than fivefold (and up to 210-fold) enrichment relative to total hippocampal homogenate (electronic supplementary material, table S1).

These are extremely high values of synaptic enrichment compared with most small RNAs as well as typical miRNAs and their precursors, which only show enrichment up to a maximum of approximately fivefold [28,30] (figure 1) even when measured using the same deep sequencing methods [41]. Almost all of the synaptically hyper-enriched sequences were 29–35 nt in length. The majority were derived from C/D box snoRNAs, but sequences were also derived from several sites within 18S rRNA, as well as one from 28S rRNA. Interestingly, one 33 nt sequence was derived from Malat1 (Neat2), a long ncRNA which regulates alternative splicing and which modulates synaptic density in neurons [85] (electronic supplementary material, table S1). This sequence is identical in mouse and human genomes. It is surprising to observe synaptic Malat1-derived RNA, because Malat1 is generally thought to reside in the nucleus. However, Malat1 is known to bind TDP-43 [86] which is expressed both in nuclear and dendritic locations, so Malat1 could possibly be one of the growing list of nuclear proteins found to have dendritic expression (see §12). Malat 1 might regulate alternative splicing locally in dendrites, and its processing to small RNAs might also contribute to its regulation of synaptic functions.

We still do not know the mechanisms by which the pincRNAs are formed, nor the targets that they regulate. However, these data indicate that a subset of this novel class of small RNAs are strongly induced during an early phase of learning, and a subset show extremely high synaptic enrichment, suggesting that they are expressed locally (and perhaps formed locally) near synapses. Clearly, this is a promising area for further investigation.

## 8. piwi-interacting RNAs in brain

The piRNAs were first discovered and characterized in the germline [87]. They comprise a heterogeneous set of sequences: typical piRNAs arise from long, single-stranded intergenic piRNA-generating transcripts that are enriched in TEs and repeat elements, and that may give rise to secondary piRNAs that have the antisense orientation via a ‘ping-pong’ processing mechanism [87]. Another class arises from unique genomic loci, particularly the 3′-UTRs of protein-coding RNAs and ncRNAs [88]. The piRNA system has a similar spectrum of activities as the endo-siRNA system: targeting TE transcripts, regulating the translation of specific cellular genes and affecting epigenetic modifications in the nucleus. Recent studies have confirmed that piRNAs are, indeed, expressed in many mammalian somatic tissues including brain [89]. In *Aplysia*, several piRNAs are modulated by serotonin, and the piwi/piRNA complex facilitates serotonin-dependent methylation of a conserved CpG island in the promoter of CREB2, the major inhibitory constraint of memory in *Aplysia*, leading to enhanced long-term synaptic facilitation [90]. The expression of piRNAs in mammalian brain is at apparently low levels, but this might be underestimated, in part, because they have 2-*O*-methyl groups added to the 3′-end which makes their detection less efficient using most current sequencing methods.

## 9. Alu-related transcripts

The first discovered ncRNA expressed near synapses is BC1 [91]: it is derived from a retroposed tRNA sequence that gave rise to genomic repeats (so-called ID elements) expressed in rodents; it is brain-specific, very abundant, modulated by synaptic activity and specifically transported to dendrites where it is highly enriched in synaptic fractions. Its expression is driven by polIII; it is brain specific *in vivo* but appears rather widely in cultured or transformed cells and is transiently induced by cellular stress. BC1 regulates translation of proteins within dendrites by binding to several different proteins [92]. Double knockouts of BC1 and FMRP in mice produce cognitive and behavioural deficits that are stronger than observed with single gene knockouts, and suggestive that they both affect the same molecular pathway(s) [93]. BC200 and G22 are primate-specific RNAs that derive from a different type of genomic repeat, Alu, yet exhibit similar dendritic targeting and function regulating translation as described for BC1 [94].

This story is familiar to neuroscientists. However, should we regard BC1 and BC200 as unrelated ‘accidents’ which arose randomly by rare genomic alterations, which just happened to regulate translation locally within dendrites? Recent studies raise the possibility that a much larger population of repeat-related ncRNAs are also involved in synaptic plasticity.

For example, consider the family of Alu-related ncRNAs [95]. Full-length cytoplasmic Alu transcripts, monomeric Alu (scAlu) and related transcripts are expressed in neural tissue. Though the Alu family is primate specific, related repeats are expressed in rodents (B1 SINEs). These are driven by polIII from multiple genomic sites; as a population, they are induced by cellular stresses such as cycloheximide treatment, heat shock or viral infection, though individual transcripts differ widely in their cell type expression and inducibility [96]. One of their actions appears to be global inhibition of cap-dependent protein translation, though multiple effects on translation have been reported [95]. Accumulation of cytoplasmic full-length Alu transcripts appears to mediate cell death in pigment epithelial cells, a process that is prevented by dicer which cleaves Alu into smaller RNA fragments (though the effect of dicer may be to destroy the ‘toxic’ Alu directly and is not necessarily mediated by the formation of typical endo-siRNAs) [97].

To my knowledge, no one has examined whether cytoplasmic full-length Alu, scAlu transcripts or pre-mRNAs containing intronic Alu sequences are actively transported into dendrites, particularly following cellular stresses. However, this is worth examining since dendritically enriched BC200 is derived from Alu, and both RNAs bind SRP9/14 and poly(A)-binding protein which are expressed in transport granules. HnRNP A2 not only binds both BC1 and BC200 [98], possibly mediating their targeting, but pre-mRNAs that contain intronic ID elements are also dendritically targeted [99]. The transport protein staufen has been shown to bind inverted Alu repeats contained within 3′-UTRs [100] as well as duplex structures formed by binding of two Alu-containing transcripts to each other [101]. One ncRNA (the miRNA precursor for miR-134) is known to be targeted to dendrites via binding to DHX36 [102]; interestingly, DHX36, hnRNRP A2 and HuR all bind certain intronic Alu sequences found within pre-mRNAs [103].

Another Alu-related transcript family is the small NF90-associated RNAs (snaRs), a primate-specific family of approximately 117 nt small RNAs that derive almost entirely from Alu sequence, form extensive intramolecular double-stranded secondary structures, are driven by polIII and are expressed in testis and other tissues including brain [104]. SnaR-A appears to have been derived from the left monomer of Alu (scAlu) and is noteworthy since BC200 is also derived from a left monomeric Alu sequence. Like monomeric scAlu, snaRs associate with both ribosomes and polyribosomes [105]. Different members of the snaR family (e.g. snaR-A versus snaR-G2) differ markedly in their relative expression among individual brain regions and across different tissues [106]. It is not clear how the functions of snaRs relate to their binding to NF90, which itself binds (and inhibits translation of) several target mRNAs bearing AU-rich response elements, as well as binding (and inhibiting the processing of) several primary miRNA gene precursors [107,108]. It will be interesting to investigate whether snaR expression is regulated by neuronal activity, whether snaRs are transported to dendrites and whether (as would be expected) they regulate protein translation.

Still another example is NDM29, an approximately 350 nt cytoplasmic transcript consisting of both Alu and unique sequences, that is driven by polIII and encoded within an intron of ASCL3 [109–111]. Its expression is induced during neuronal differentiation, and transfecting NDM29 into undifferentiated neuroblastoma cells causes both differentiation into a neuron-like phenotype and reduction of cell growth and malignancy.

There are several ways in which Alu-related transcripts may regulate mRNAs. The first report describing cytoplasmic B1 SINE transcripts noted that it derived from an intron of a protein-coding gene in antisense orientation and proposed that it may regulate the host gene via sense–antisense interactions [112]. A genome-wide examination of predicted polIII transcripts shows that the majority reside within introns in the antisense orientation to the host gene, which may regulate alternative splicing of the host gene [113]. Alu and B1 repeats show strong bias towards retention of repeats in the antisense strand of introns [114,115]. Repeats expressed in sense orientation are associated with different functional GO categories of mRNAs than those expressed in antisense orientation, suggesting that they may preferentially target certain molecular pathways [114–116]. Krichevsky *et al.* [116] noted that differentiation of human HL-60 cells is accompanied by the rapid induction (and association with polyribosomes) of a long ncRNA that contains two Alu repeats in antisense orientation. Its induction is accompanied by a shift into polyribosomes of a population of mRNAs containing Alu sequences in their 3′-UTRs.

A variety of reports have shown that both small and long ncRNAs can regulate target RNAs via repeat sequences which are embedded within 3′-UTRs or other non-coding regions [101,117–119]. Alu- and B1-related transcripts are attractive regulators because (like miRNAs) a single transcript can target potentially hundreds of mRNAs (that express the same repeat or repeat fragment in the opposite orientation). Intriguingly, many miRNAs target a conserved site within Alu and B1 repeats in sense orientation [120]. Although most Alu sequences embedded within mRNAs do not show optimal features for being regulated by miRNAs [121], they do create functional miRNA target sites in a significant minority of cases [122].

These observations strongly suggest that Alu repeat-related transcripts comprise a novel class of translational regulators in neurons (and likely in dendrites). Although full-length cytoplasmic Alu transcripts are thought to show very low expression in most cells under resting conditions, cytoplasmic Alu transcripts have been shown to be induced by glucocorticoids in liver cells [123] and by retinoic acid in embryonic stem cells [124], so they might potentially play physiological roles in neurons under some defined situations. Alu-related transcripts could also potentially play a role in deficits observed following cellular stresses in neurons. For example, Alzheimer disease mouse models and human brain tissue exhibit hallmarks of cellular stress (i.e. increased phosphorylation of eIF2α), and Alzheimer disease cortex shows an upregulation of BC200 relative to age-matched controls [125] as well as an upregulation of NDM29 [126]. If ‘toxic’ Alu [97] were to be targeted to synapses, that might disproportionately damage the synaptic compartment and contribute to the pathogenesis of this disease.

## 10. Other transposable element-related transcripts

Besides the family of Alu-related repeats, other classes of TE transcripts, including LINEs, SINEs and LTRs are also expressed in mammalian brain during development and in maturity [127]. Most discussions of cytoplasmic TE transcripts have assumed that piRNAs and endo-siRNAs have the job of fighting and destroying them so that they will not transpose into the genome. Indeed, somatic transposition of LINE-1 elements can occur into the genome of neuronal progenitor cells, which may increase neuron-to-neuron variation in gene expression and may have adverse effects in aging and neurodegenerative diseases [127]. ‘Toxic’ RNA repeats can cause neurodegeneration [128–130] potentially via several mechanisms, including binding cellular proteins needed for health, competing for dicer-dependent processing of miRNAs and activating Toll receptors.

However, particularly in a post-mitotic cell type such as the mammalian neuron, which does not appear to support transposition, TE transcripts could potentially acquire benign regulatory functions of their own within the cytoplasm. TE sequences are embedded in both introns and 3′-UTRs of many mRNAs, where they serve as targets for certain miRNAs [122,131], piRNAs and endo-siRNAs, and might also be targeted by TE-related transcripts. Li *et al*. [132] have recently reported that a broad sampling of transcripts from many TE families (including Alu and other SINEs, LINEs and LTRs) are expressed in normal human brain and are tightly associated with TDP-43. This is compatible with a physiological role for these transcripts, and might provide a mechanism for their transport to dendrites.

To my knowledge, no one has examined whether any cytoplasmic TE transcripts are induced following physiological levels of neuronal activity, or whether they show preferential transport to dendrites. However, in view of the Alu-related family of transcripts already discussed, and in view of theoretical considerations (discussed in §11), it is worth examining L1, L2 and other families of TE-related transcripts to learn whether they have physiological roles in neurons.

## 11. Why do non-coding RNAs tend to be non-conserved across species?

The basic protein machinery of synaptic transmission is highly conserved throughout evolution, with many synaptic genes being found even in sponges [133]. Processes of synaptic development and neurotransmission are remarkably similar from *C. elegans* and *Drosophila* to mouse and man. By contrast, the individual ncRNAs associated with synapses tend to be non-conserved across species. In the case of miRNAs, we previously pointed out that the subset of mouse hippocampus miRNAs that are significantly enriched in synaptic fractions tend to be evolutionarily new, with many found only in mammals or only in rodents [30], in contrast to non-enriched miRNAs that tend to be more broadly conserved and expressed in many tissues. How can we reconcile this with the notion that they play essential roles in synaptic plasticity?

The species specificity of ncRNAs is not limited to miRNAs, but affects the entire world of ncRNAs. For example, none of the hairpin endo-siRNAs encoded within introns of synaptic genes were conserved between mouse and man [76] and, in general, intronic hairpins tend to be species specific [134]. Sense–antisense gene pairs show relatively little conservation between mouse and man [135]. Repeat-derived transcripts such as BC1, BC200, G22 and Alu-related repeats show limited, lineage-specific expression across evolution. The piRNAs also show little conservation from mouse to man or even across related primate species; they even show appreciable changes across human populations [136]. How should we interpret this?

One possible interpretation is that non-conserved ncRNAs are simply not functional, or at least their functions do not confer any selective advantage which would cause positive selection. This is compatible with a prevailing model wherein miRNAs arise randomly (and frequently) within genomes: all that is needed is some minimal level of transcription, and a stem–loop secondary structure that can be processed by drosha, dicer or other enzymes [137]. Such nascent miRNAs would be expected to be driven haphazardly by nearby transcriptional control elements and would tend to show expression at low levels and perhaps only in a few tissues. Nascent miRNAs that do not acquire positive functions exhibit random sequence drift and are quickly lost in evolutionary time [137].

However, much of the data are compatible with the opposite scenario: that certain ncRNAs show accelerated positive evolution, and in fact, may change so quickly that their relation to homologues in other species becomes obscured. Among protein-coding genes, this effect is best documented for genes involved in brain growth and synaptic function, and among ncRNAs, this is best documented for piRNAs [138]. Species-specific differences in piRNAs and other ncRNAs appear to contribute to the setting up of species barriers to reproduction [138]. Conversely, a mouse species-specific dicer isoform has acquired regulatory activities in oocytes that are actually essential for reproduction [139].

The ncRNAs are quite diverse (miRNAs, repeat-derived miRNAs and transcripts, hairpin endo-siRNAs, antisense RNAs and piRNAs), yet they all demonstrate a general principle that the ncRNA sequences do not need to have any intrinsic ‘meaning’ or function in order to exert important regulatory effects. Rather, these ncRNA sequences acquire value by virtue of having complementarity to other sequences (residing within the precursor or host gene in *cis*, or within other transcripts in *trans*) [140]. Often the complementarity remains functional in the face of a few base mismatches. Often the sense and antisense sequences arise from the same chromosomal locus (or from the same TE inserted into multiple loci) so that genetic changes in primary gene sequence that occur will affect both sense and antisense sequences in parallel, thus preserving the complementarity. These features may explain, in large part, why ncRNAs differ so dramatically from protein-coding genes, both in terms of the usual indicators of functionality and in terms of evolutionary constraints.

In fact, it can be argued that a complementarity-based system works best when the sequences involved are otherwise totally arbitrary and self-contained, for then they will minimize off-target effects! This is one reason that I find it attractive to consider that TE transcripts and TE-derived small RNAs may comprise a primordial system of computational elements, of which miRNAs, endo-siRNAs and piRNAs represent specializations. Using the term ‘computational elements’ emphasizes not only that ncRNAs have biological functions, but that they respond to contextual, nonlinear and interactive influences that make the output more than a simple function of the input. To give just one example, a given miRNA may be inhibitory, ineffective or actually enhance translation of a target mRNA, depending on what other proteins bind nearby on the mRNA and what post-transcriptional modifications they bear as a function of the cell cycle [141].

The fact that ncRNAs do not appear to be essential for neurotransmission is not necessarily a bad thing, and paradoxically, may be a clue to their importance. Separating the regulatory system from the nuts-and-bolts of the synapse allows ncRNAs to evolve more freely. Synapses are not simply describable as being in activated, resting or depressed states, but are simultaneously regulated by ncRNAs which control their potential responses to new stimuli (a form of metaplasticity; see also [142,143]).

## 12. Long non-coding RNAs

Given their number and diversity, long ncRNAs must undoubtedly be important regulators of brain functions. Mercer *et al.* [144] have catalogued long ncRNAs that are expressed in dendrites, and Lipovich *et al.* [145] have carried out a genome-wide analysis of long ncRNAs that are modulated by neuronal activity in human brain. Long ncRNAs can act as miRNA sponges, and can bind proteins and RNAs that regulate transcriptional changes and epigenetic modifications of chromatin. A few types of long ncRNAs have been localized near synapses; for example, pre-mRNAs with retained introns [24], sense/antisense transcript pairs [77], pri-miRs [28,146] and natural antisense and other ncRNAs that are selectively transported via kinesin in *Aplysia* [147].

## 13. Coordinating dendritic events with transcriptional and epigenetic mechanisms in the nucleus

A variety of proteins generally thought of as being selective nuclear components have been shown to be expressed in dendrites, among them transcription factors, transcriptional co-activators and RNA-binding proteins. As discussed in §3, RNA-binding proteins and splicing factors may mediate dendritic RNA transport and process pre-mRNAs within dendrites. Spikar is a transcriptional co-activator that is expressed in the nucleus as well as within dendrites. Interestingly, extra-nuclear spikar binds the spine protein drebrin and regulates spine formation in a drebrin-dependent manner, suggesting that it may have local actions within dendrites [148].

Dendritic transcription factors and co-activators are also part of a larger system in which events occurring near synapses are communicated back to the nucleus, to be coordinated with transcriptional and epigenetic modifications that mediate long-lasting changes in gene expression which are necessary for memory formation and persistence [149]. Many transcription factors and co-activators are known to be translocated from dendrites to the nucleus in an activity-dependent manner [150]. A partial list includes CREB [151], NFκB [152], Jacob, importin-α [153], CRTC1 [154], AIDA-1d [155] and abi-1 [156]. In the case of Jacob, modifications to the protein differ according to whether extrasynaptic or synaptic NMDA receptor activation is elicited [157]. Thus, there is some degree of local dendritic information conveyed back to the nucleus, even if all Jacob molecules are transported back to a single destination.

Might synaptic ncRNAs participate in synapse-to-nucleus signalling or vice versa? So far there are only a few clues. Entry of cytoplasmic siRNAs into the nucleus is necessary for a behavioural adaptation to occur in *C. elegans* [74]. As well, dicer fragments generated by synaptic activity bear nuclear import sequences and are potentially translocated to the nucleus [29]. Conversely, miRNAs might potentially route newly transcribed RNAs coming from the nucleus into specific synaptic destinations: in this scenario, miRNAs that are locally produced near activated synapses could bind to, and thus preferentially trap, newly synthesized mRNAs that are transported down dendrites. This might keep them in close proximity to the previously activated synapse, in a state of tonic inhibition, until a subsequent stimulus de-represses the miRNA influence and allows local translation of the mRNA to occur [30].

## 14. Dendritic mitochondria

Mitochondria modulate both presynaptic and postsynaptic transmission via regulating local calcium, redox and ATP levels, and play at least permissive roles in dendritic plasticity [158]. Their motility is inhibited by synaptic activity, and individual mitochondria can be trapped or anchored to individual dendritic branches or spines [159].

Insofar as mitochondria have their own distinctive protein synthesis mechanisms (of the prokaryotic type), one should consider whether ncRNAs might be regulating targets related to translation within dendritic mitochondria. Mitochondria express several mRNAs and tRNAs, as well as small RNAs [160], antisense transcripts [161] and several mitochondrially encoded small RNAs which are strongly induced during olfactory discrimination learning [82]. A number of nucleus-derived miRNAs and Ago2 are associated with mitochondria [162,163]. Inhibitors of prokaryotic protein synthesis, which block mitochondrial translation selectively, have been reported to inhibit learning [164].

Moreover, let's not forget that mitochondria have their own genomes! Transcription factors have been found within mitochondria, and in particular, neuronal mitochondria contain CREB which binds directly to CRE within the mitochondrial genome and regulates transcription of mitochondrial genes [165,166]. Although mitochondrial DNA lacks histones, mitochondrial DNA does become epigenetically modified by both 5-methylcytosine and 5-hydroxymethylcytosine; the latter is regulated during aging [167] and in response to valproate [168]. Thus, it is conceivable that dendritic mitochondria participate in learning and memory, in part, by providing a portable genome that is locally regulated both transcriptionally and epigenetically.

## 15. RNA transfer

So far, this discussion has assumed that proteins, mRNAs and ncRNAs are transported into dendrites for the purpose of functioning locally near synapses. Yet, following the discovery that secretory exosomes contain RNAs [169], there is growing awareness that these molecules can be packaged and transferred in an activity-dependent manner from cell to cell. The RNA transfer field is exploding (not unlike the field of ncRNAs!) and several recent reviews have summarized the current state of evidence for vesicular transfer of proteins and RNAs among cells in the central nervous system ([170–176] and this volume). Here, I shall discuss only a few points that are relevant to RNAs as computational elements for synaptic plasticity.

(i) *Synaptic spinules* are little finger-like protrusions [177] that form at the postsynaptic face of the dendritic spine (adjacent to the PSD) in response to depolarization or NMDA receptor activation, leading to elevated intracellular calcium levels. Spinules protrude into neighbouring presynaptic terminals and glial cells where they are engulfed and pinched-off by clathrin-coated endocytosis [178,179]. The process is rapidly induced (within a minute) and reversed when the depolarization is removed [180]. Although the nature of their cargo is unknown, almost certainly synaptic spinules transfer membrane proteins as well as RNAs and other cytoplasmic contents relating to the region immediately adjacent to the synapse [170]. Because this region expresses synaptic mRNAs, miRNAs and pri-miRs [28,30], it is likely that these RNAs are among the cargo, and this region expresses eIF4E and other proteins related to translation as well [8]. The original report of spinules said that ‘ribosome-like particles are frequently present in the vicinity of the spine apparatus and within the cytoplasm of the spinule’ [177], so it is possible that spinules contain most, if not all, of the machinery necessary for protein translation. The biology of synaptic spinules shows intriguing parallels with that of secretory exosomes [170].

Protein synthesis is known to be important for growth, branching and targeting of axonal growth cones during development [181], but is thought to occur only at very low levels in presynaptic terminals in the mature brain. This has been seen as an objection against the idea that synaptic mRNAs could serve as retrograde messengers [182]. However, if both mRNA and the protein machinery for translation are transferred from the postsynaptic neuron via spinules, this would allow for presynaptic translation to occur following high synaptic activation. Even a small amount of mRNA could have a high relative impact when the system is otherwise inactive. Transfer of proteins such as CAMKIIα and α-amino-3-hydroxy-5-methyl-4-isoxazolepropionic acid (AMPA) receptors may also contribute to presynaptic plasticity [170].

The spinule contents that are being transferred from a given dendritic spine need to be replenished by activity-dependent transport from the cell body, or at least from nearby sites on the dendritic tree, lest the transfer process result in synaptic depression due to loss of postsynaptic components. Thus, the synaptic outcome should be quite different for a single spine depolarized in isolation versus a spine that is depolarized at the same time as the entire postsynaptic neuron. Jobe *et al.* [183] proposed, on theoretical grounds, that transfer of synaptic mRNAs (and polyribosomes) to the presynaptic terminal would lead to new axonal sprouting and synapse formation. Their model predicted different outcomes depending on whether a given dendritic spine is activated at the same time as the rest of the postsynaptic neuron (i.e. whether it coincides with its firing), and whether or not a reinforcement input is present at the same time as well. They argued that such a process could mediate the cellular equivalent of ‘backpropagation’ discussed in neural network models and could account for both Hebbian and non-Hebbian changes in synaptic efficacy [183].

(ii) *Secretory exosomes*, or exosomes for short, are derived from multivesicular bodies within endosomes [184]; exosome-sized vesicles also can originate via an alternative pathway that buds directly from the plasma membrane. Exosomes are rich in proteins that are related to translation (e.g. ribosomal subunits and elongation factors); they express specific sets of mRNAs, miRNAs and other ncRNAs including TE-derived transcripts, and they express cell-specific membrane markers that can be used for selective targeting. Thus, they appear ideal as a means for cells to modulate protein translation within their recipient targets [184].

Although most studies have focused on the transfer of mRNAs and miRNAs, the mammalian system of vesicular communication is also related to the RNAi systemic gene silencing system studied in *C. elegans* and plants. Certain types of mammalian neurons express SID-1 [2], which acts as an RNA-permeant pore for siRNAs and double-stranded RNAs, though little is known yet regarding its subcellular localization or its possible roles in neurons. Mammalian cells load siRNAs on RISC in proximity to multivesicular bodies [185], and exogenous siRNAs are packaged efficiently into exosomes which can mediate RNA silencing in recipient cells [186].

As in the case of synaptic spinules, the secretion of neuronal exosomes is greatly stimulated by depolarization or NMDA receptor activation, leading to elevated intracellular calcium levels [187,188]. Cultured cortical neuron exosomes express L1-CAM, AMPA receptors (GluR2/3) and prion protein, but not NMDA receptors or PSD-95 protein [187]. Moreover, exosomes can be released from sites within the dendritic tree [188], suggesting that local packaging and release of dendritic molecules may occur. Release of exosome-like vesicles from presynaptic terminals has been documented at the *Drosophila* neuromuscular junction, where it is thought that they are secreted at the sides of the active synapse [175]. Neuronal exosomes express miRNAs [172]; however, it is not well understood how exosome release or miRNA secretion [189] may be related to exocytosis of synaptic vesicles during neurotransmission.

In any case, the majority of exosomes released from somatic and dendritic regions may be expected to communicate non-synaptically, in a ‘sideways’ manner, with adjacent neurons, interneurons and glial cells. Because there is so little free extracellular space in the brain, it is likely that neuronal exosomes secreted *in vivo* would be taken up predominantly by immediate neighbours. Neurons in the cortex are organized into minicolumns, which tend to show highly correlated input and output firing [190]. If neighbouring neurons that are activated together release exosomes together, this may provide a means to ‘synchronize’ their gene expression. This, in turn, may help establish or reinforce a circuit-level memory representation that is retained by the minicolumn as a whole.

## 16. Summary and conclusion

The current state of knowledge is incomplete and even fragmentary in many ways. However, it is clear that members of many, and perhaps all, of the known classes of ncRNAs are expressed locally (and may be processed locally) within dendrites and within dendritic spines. The miRNAs and other ncRNAs provide another layer of regulation on top of the mRNA system (controlling the transport, splicing, localization and translation of synaptic mRNAs). Together, they support an enormous increase in information capacity as compared with a single pattern of gene expression per neuron. ncRNAs differ dramatically from protein-coding genes, both in terms of the usual indicators of functionality and in terms of evolutionary constraints. They do not appear to be essential for neurotransmission to occur, yet are crucial for orchestrating synaptic plasticity; and may help drive changes in cognition that are species-specific (including the case of human brain evolution).

Looking ahead to the next decade, I predict that four nascent areas of investigation will become more intensified and more connected to the mainstream of neuroscience:

First, the notion of the dendritic spine/dendritic branch as a quasi-independent computational unit, which is currently accepted in neurophysiology, will be extended to cell biology, as more and more functions thought to be restricted to the nucleus turn out to play local roles within the synaptic compartment. Evidence is already strong for alternative splicing of pre-mRNAs and local biogenesis of miRNAs. As pointed out, it is conceivable that RNA editing of pre-mRNAs may occur locally, as well as transcriptional regulation and epigenetic modifications of dendritic mitochondria. Second, ncRNAs will be shown to contribute widely to synaptic plasticity in mammalian brain via local biogenesis of synaptic miRNAs, RNAi (mediated by endo-siRNAs and piRNAs) and possibly novel mechanisms (e.g. pincRNAs). Third, Alu-related and other TE transcripts will be shown to have important physiological and pathological roles within neurons, independently of their transposition into the genome. Fourth, our understanding of information processing in the brain will be transformed by the recognition that neurons transfer RNAs across synapses and to their neighbours via synaptic spinules, secretory exosomes and possibly other mechanisms (e.g. RNA-permeant pores).

A century after Cajal formulated the Neuron Doctrine, we still know little of the mechanisms that trigger or store memories in the brain, and these latest findings provide but a few more pieces to the puzzle. Nevertheless, I think we are moving in the right direction!

## Supplementary Material

Supplementary Table S1
